# Microarrays for Microbes: the BμG@S Approach

**DOI:** 10.1002/cfg.187

**Published:** 2002-08

**Authors:** Jason Hinds, Kenneth G. Laing, Joseph A. Mangan, Philip D. Butcher

**Affiliations:** Bacterial Microarray Group Department of Medical Microbiology St. George's Hospital Medical School Cranmer Terrace, Tooting London SW17 0RE UK


**Comparative and Functional Genomics**
*Comp Funct Genom* 2002; **3**: 333–337.
Published online in Wiley InterScience (www.interscience.wiley.com). **DOI:** 10.1002/cfg.187

**Conference Review**

### Microarrays for microbes: the BμG@S approach

Jason Hinds,* Kenneth G. Laing, Joseph A. Mangan and Philip D. Butcher
*Bacterial Microarray Group, Department of Medical Microbiology, St. George's Hospital Medical School, Cranmer Terrace, Tooting, London SW17 0RE, UK*

**Correspondence to: Jason Hinds, Bacterial Microarray Group, Department of Medical Microbiology, St. George's Hospital Medical School, Cranmer Terrace, Tooting, London SW17 0RE, UK. E-mail: j.hinds@sghms.ac.uk*

Received: 10 June 2002
Accepted: 14 June 2002

**Keywords:** microarray; bacterium; pathogenesis; functional genomics

### Introduction

The Bacterial Microarray Group at St George's Hospital Medical School (BμG@S; **http://bugs.sghms.ac.uk**) has been funded by The Wellcome Trust as part of the Resources for Functional Genomics Initiative. A 5 year programme grant entitled 'A Multi-Collaborative Microbial Pathogen Microarray Facility' was awarded to Dr Philip Butcher and Joseph Mangan (St. George's Hospital Medical School), Professor Brendan Wren (London School of Hygiene and Tropical Medicine), Professor Neil Stoker (Royal Veterinary College, London) and Dr Keith Vass (Beatson Institute/Glasgow University). The aim of this funding is to enable a community of collaborating researchers with a common interest in bacterial pathogenesis to have rapid, flexible and cost-effective access to current microarray technology. Rather than each individual group having to invest in the equipment and the lengthy learning and optimization process involved in microarray set-up, the aim was to centralize the expertise within one group that would then be made freely available to collaborating groups as a functional genomics resource. The project was funded to produce whole-genome DNA microarrays for 12 bacterial pathogens within 2 years and provide training and support in the use of the arrays over a period of 5 years. This article provides an overview of the project organization, the technology and support involved in producing and using the microarrays, future developments and a summary of the progress to date.

### Project structure

The ethos behind the project was to create a collaborative network of scientists who would all interact with both the central microarray facility (BμG@S) and other groups within the network. Rather than the arrays being produced on a 'commercial' basis and simply handed out to 'customers', the aim was to create working collaborations in which BμG@S scientists and pathogen researchers would pool together their respective areas of expertise to advance the area of bacterial functional genomics rapidly and in a more focused manner. The collaborative network currently consists of over 30 academic research groups, including those interested in particular bacterial pathogens, aspects of pathogenesis,

Copyright © 2002 John Wiley & Sons, Ltd.

genome biology, bioinformatics or the analysis and management of microarray data.

For a large collaborative project of this nature, the organizational structure is important to ensure that the communication between groups is clear and that the process is transparent and fair for all users. The BμG@S group form the central hub of this network and has scientists funded to design and construct the arrays, provide training to users, supply informatics support in the form of databases and analysis tools and also give general administrative support to the project. Around this core facility the user groups, clustered by bacterial pathogen and array, provide expert knowledge of each pathogen to aid the design process and also coordinate the use of the arrays. The user community decide the best use of the arrays available, rather than the central facility. Overseeing the whole process is the steering committee, which is primarily composed of independent scientists and the grant holders, as well as representatives from the BμG@S group and The Wellcome Trust. The purpose of the steering committee is to provide independent and impartial advice, monitor progress and deal with any issues arising from the project. The collaborating groups form a network around this central structure of BμG@S, user groups and steering committee, interacting not only with BμG@S but also with each other. New research groups wishing to access the BμG@S arrays do so on the basis of agreed academic collaboration with one or more of the existing pathogen user-groups. Collaborating groups include biologists using the arrays in their research, genome sequencing centres, such as The Wellcome Trust Sanger Institute, groups involved in data analysis and other independent research-based array facilities.

### Microarray design

The microarrays produced by our group are of the spotted PCR product type, generally based on protocols originally developed by the Brown lab in Stanford University (**http://cmgm.stanford.edu/pbrown/**) and used by many groups [2,3]. The first stage in the process is the design of the microarray. The basic requirement for a microarray intended for a global genome-wide analysis is to have each gene in the genome represented on the array. The starting point for any array is commonly an annotated genome sequence that provides a defined reference in terms of gene predictions and annotations. By working closely with the user groups for each pathogen, any important biological features, identified from either the genome sequence or expert knowledge of the pathogen, can be incorporated in the array design. For example, information about unusual gene families or hypervariable genes can be accounted for in the design process to ensure that the maximum amount of information can be gained for each gene. The inclusion of the user community at the design stage, combined with the flexibility that is inherent with in-house array production, enables the arrays to be customised to meet particular needs. Additional elements may be added to the array to represent genes not present in the sequenced strain, possibly identified in other strains by methods such as subtractive hybridization, or alternatively to include elements to represent related genes from other bacterial species that are considered useful.

Increasingly, there are genome sequences available for numerous strains of a particular bacterial species, or even closely related species. It is therefore important that the array design should cover all available genome sequences of a species, so that composite arrays are constructed, either at the outset in the initial design or at a subsequent stage in the continuing development and evolution of an array, as more sequences become available. The majority of genes are common to all strains and have a high degree of homology between strains; this means that an element on the array which represents a gene in one strain will also represent the homologous gene in other strains. Therefore, the production of a composite array would require an element to represent each of the genes common to all strains plus the strain-specific genes and we have developed methods to achieve this gene selection.

The spotted PCR product approach employed means that the overall objective of the design process is to generate gene-specific primer pairs to enable amplification of a PCR product to represent each of the genes selected for inclusion on the array. Software has been developed so that, essentially, a whole-genome sequence is input at the start and the primer sequences are output at the end. The basic stages in this process are to extract the gene sequences and systematic identifiers from an annotated genome sequence file,

Copyright © 2002 John Wiley & Sons, Ltd. 

design several possible PCR products for each gene using Primer3 [4] and then select the most suitable PCR product to represent each gene. The PCR product selection is important, as it is primarily based on BLAST [1] analysis of the potential PCR product sequences against the gene sequences, and thus highlights any likely cross-hybridization by non-target genes. Ideally, the PCR product selected should be unique to the target gene, but if this is not possible it should have the minimum amount of cross-hybridization to the other non-target genes. This selection approach helps maintain data clarity by ensuring gene-specific signal for the maximum number of genes.

### Microarray construction

Following the design stage, the next step in the process is the generation of the PCR products that will form the elements on the array. The oligonucleotide primer pairs are supplied in a 96-well format compatible with the robotics used for PCR. All liquid handling and PCR cycling for generation of the PCR products are conducted using a RoboAmp 4200 (MWG Biotech) that incorporates a non-cross-contamination design, so that only a single well is open at any one time during the process. Whilst around 90–95% of the PCR products are obtained at the first attempt, there then follows a process to complete all the PCR products. This involves repeating reactions, modifying reaction conditions and possibly resynthesizing the oligonucleotide primers. The PCR products are all verified by agarose gel electrophoresis to ensure that a single product of the correct size is obtained and further verification is achieved by sequencing 5% of the PCR products. Clearly, it would be favourable to sequence verify all products, although this would substantially add to the cost of construction. However, an aliquot of the PCR product that forms the array element is retained, so that users can verify the sequence of the element at a later date to validate any interesting results, thus furthering the sequence verification of the array.

Having successfully completed all the PCR reactions, there then follows the preparation of the PCR products for printing the array. Purification and concentration of the PCR products is achieved by precipitating and washing an aliquot of the PCR reaction and resuspending in a reduced volume of 50% DMSO for printing. The PCR products are transferred to 384-well plates for printing and spotted at high density on poly-L-lysine-coated glass microscope slides, using a MicroGridII (BioRobotics) arraying robot. The capacity of the robot allows it to print to a maximum of 108 glass microscope slides from a total of 24 384-well plates. Split-pin technology used in the arraying robot allows a spot to be deposited on a total of 108 slides from a single visit to the PCR products, generally producing spot size of 150–180 μm. To give some appreciation of the scale, a microarray for a bacterial genome is often printed in duplicate in an area of 20 × 20 mm. In addition to elements that represent the bacterial genome, a number of control elements are also spotted on to the array. Typical controls include bacterial ribosomal RNA genes as a positive control, human genes as a negative control or for use with spiking experiments, controls to check for carry-over of samples during printing and also fluorescently labelled oligonucleotides that act as orientation markers or 'landing lights' on the array.

The quality of the printed arrays is assessed in a number of ways before they are used experimentally. Scanning a sample of the arrays directly after printing gives some indication that the arraying has proceeded successfully, highlighting any gross failures of the pins or spotting artefacts. After the slides have been post-print processed, undertaken to attach the DNA to the slides and block any non-specific binding sites, the DNA on the arrays may be stained with a fluorescent dye or test hybridizations performed to check that the DNA is still present and attached to the arrays and also confirm the absence of any spots due to pin 'misfiring' during printing.

### Application of microarrays

The finished microarrays are then made available to the pathogen user groups for use in their experiments. The arrays produced present a limited resource and so the user groups for a particular pathogen ensure that the best use is made of the arrays, achieved by prioritizing the usage and avoiding unnecessary duplication of experiments. Rather than having competing groups within the multi-collaborative network attempting to perform identical experiments, the aim is to encourage

Copyright © 2002 John Wiley & Sons, Ltd. 

new collaborations that address common goals. The other conference reviews presented in this issue give a useful overview of the widespread applications that the current arrays are being used for in the investigation of the various aspects of bacterial pathogen biology.

Another strategy to ensure that users make the best use of the arrays from the start is tackled by the provision of training courses by BμG@S. The aim of the training is for users to generate good quality data from the outset, rather than having to waste arrays and time developing or optimizing protocols. The training encompasses hands-on instruction in hybridization protocols, image and data analysis software, as well as consideration of sample preparation and experimental design. The idea is for new users to gain full benefit from the expertise built up within the BμG@S group over a period of time, so the progress of individual projects is faster. By complementing the array expertise within the BμG@S team with the pathogen expertise of the users, the benefits of this multi-collaborative approach are evident. The two-way relationship of the users with BμG@S is extremely useful, as it allows the dissemination of new ideas and techniques throughout the whole network of researchers.

### Future developments

Whilst the priority of the project remains to provide microarrays based on the robust and established methods described previously, there is also the need to investigate and evaluate developing array technologies. There has been a dramatic increase in the availability of new products, reagents and approaches within the past year; some may offer clear advantages over the current methods or technologies, whereas others may not. There remains the balance between continuing with current methods that work reliably and cost-effectively and considering possibly improved but unproven approaches.

As many groups have now progressed to the stage of generating good quality array data, the problem of data management and analysis presents the next challenge. Whilst basic training and support is provided by BμG@S in the use of software such as GeneSpring (Silicon Genetics) for data analysis and visualization, there is the need to develop more customized analysis approaches and tools. Through collaborations with groups that have an interest in developing methods for microarray data analysis (see Wernisch, p. 372 and Wolkenhauer *et al.*, p. 375), such tools will be established. The ultimate aim is to integrate the analysis approaches developed by these groups into a database (see Witney and Hinds, p. 369) that will allow users to gain access to array information, experimental data and analysis tools on a common platform.

### Current progress

At this point in the project whole genome arrays have been completed for *Campylobacter jejuni*, *Mycobacterium tuberculosis*, *Haemophilus influenzae* and *Yersinia pestis*, as well as arrays for 500 selected genes in *Streptococcus pneumoniae* and the plasmid genes of *Salmonella typhi* and *Yersinia pestis*. Each of the arrays undertaken have benefited from the ability to generate custom arrays that can be modified to a particular requirement determined by the users. For example, both the *H. influenzae* array and the latest version of the *C. jejuni* array include additional genes not present in the sequenced strain, discovered either in sequence databases or by experimental investigation, making the arrays more inclusive for a greater number of strains. It should be acknowledged that whilst The Wellcome Trust have funded the majority of the arrays produced and planned within the project, there has also been external funding from collaborators, which is detailed in the acknowledgements.

Whole genome arrays currently in progress, either in the design or construction stage, are for *Streptococcus pneumoniae*, *Staphylococcus aureus* and *Neisseria meningitidis*. The remaining microarrays outstanding under the current funding are for *Bordetella pertussis*, *Clostridium difficile*, *Chlamydia* spp., *Helicobacter pylori*, *Listeria monocytogenes* and *Mycobacterium* spp., which are planned for completion within the next 18 months.

### Summary

This report has outlined the approach taken by the BμG@S group in generating whole-genome DNA microarrays for a number of bacterial pathogens.

Copyright © 2002 John Wiley & Sons, Ltd. 

As more of these 'big biology' projects are funded, it is important to recognize the requirement to underpin these with the appropriate organizational framework to ensure effective access by the academic research community. BμG@S as a multi-collaborative resource for bacterial functional genomics provides one approach towards these goals.

### Acknowledgements

For a project of this scale there are many acknowledgements: to The Wellcome Trust for funding the BμG@S facility and all the members of the BμG@S team — Sally Husain, Richard Stabler, Gemma Marsden, Adam Witney, David Bakewell and Ceridwen Davies; Dr J.K. Vass (Beatson Institute/Glasgow University) for developing the primer design software; Dr M.J. Colston (National Institute for Medical Research) for Medical Research Council funding of the *M. tuberculosis* array; Dr P.R. Langford (Imperial College) for The Wellcome Trust funding of the *H. influenzae* array; Dr R.W. Titball (Defence Science and Technology Laboratory) for DSTL funding of the *Y. pestis* and *Y. pestis/S. typhi* plasmid arrays; Professor T.J. Mitchell for EU funding of the *S. pneumoniae* 500 array; and finally, to our commercial collaborators, BioRobotics and MWG Biotech, for their continued support throughout the project.

### References

1. Altschul SF, Madden TL, Schaffer AA, *et al.* 1997. Gapped BLAST and PSI-BLAST: a new generation of protein database search programs. *Nucleic Acids Res* **25**: 3389–3402.
2. Diehl F, Grahlmann S, Beier M, Hoheisel J. 2001. Manufacturing DNA microarrays of high spot homogeneity and reduced background signal. *Nucleic Acids Res* **29**(7): E38.
3. Hegde P, Qi R, Abernathy K, *et al.* 2000. A concise guide to cDNA microarray analysis. *Biotechniques* **29**(3): 548–562.
4. Rozen S, Skaletsky HJ. 1998. Primer3: **http://www-genome.wi.mit. edu/genome_software/other/primer3.html**.

Copyright © 2002 John Wiley & Sons, Ltd. 
